# Re-evaluating Intra-Islet Paracrine Signaling: Precision, Pulsatility and the Path toward Mechanistic Clarity

**DOI:** 10.37871/jbres2129

**Published:** 2025-06-20

**Authors:** Alejandro Tamayo-Garcia, Dayleen Hakim-Rodriguez, Rayner Rodriguez-Diaz

**Affiliations:** University of Miami, Miller School of Medicine, Department of Medicine, Division of Endocrinology, USA

## Abstract

The pancreas regulates glucose homeostasis through the rhythmic secretion of insulin and glucagon into the portal circulation-an essential process that is disrupted early in the pathogenesis of type 2 diabetes. While the metabolic relevance of this pulsatile hormone release is well recognized, the underlying regulatory mechanisms remain incompletely understood. This review highlights emerging insights that redefine pancreatic islets not merely as hormone-producing cell clusters, but as integrated oscillatory networks, capable of coordinating hormone output via tightly controlled intraislet paracrine signaling.

We emphasize the critical role of cell-to-cell communication-including interactions between endocrine and non-endocrine cells-in shaping the timing, amplitude, and composition of hormone pulses. Recent findings demonstrate that these intra-islet signals establish systemic glucose thresholds in both mice and humans, thresholds that delineate normoglycemia, prediabetes, and diabetes. Despite their clinical relevance, these mechanisms remain underexplored.

We discuss conceptual advances such as Post-Inhibitory Rebound (PIR) responses and propose that systemic hormone pulsatility emerges from coordinated activity across endocrine, neural, and vascular networks. Additionally, we address experimental limitations including receptor desensitization, ligand promiscuity, and artifacts introduced by islet isolation and static incubation assays, which lack the temporal resolution to capture dynamic paracrine interactions.

To advance this field, we advocate for the adoption of high-resolution perifusion systems and live-cell biosensor imaging. These technologies offer integrated spatial, temporal, and functional insights that are essential for uncovering the mechanisms governing hormone pulsatility and its dysregulation in diabetes.

## Introduction

### Why pulsatility and paracrinicity deserve renewed attention

The precision and coordination of hormone secretion from the endocrine pancreas-most notably the pulsatile delivery of insulin and glucagon into the portal vein-remains one of the most sophisticated yet persistently underappreciated regulatory mechanisms in systemic glucose homeostasis [1–3]. These hormones are released in coordinated oscillations approximately every 4-10 minutes, delivering rhythmic signals directly to the liver that enhance insulin sensitivity and prevent receptor desensitization. Underlying this dynamic is a network of intra-islet paracrine signaling, wherein α, β, and δ cells communicate locally through the release of hormones and other factors to fine-tune each other’s activity in real time. Although widely accepted as essential for the maintenance of euglycemia, the physiological processes that underlie this tightly orchestrated secretion-particularly those involving intra-islet paracrine signaling-remain inadequately explored and, in many respects, poorly characterized.

Emerging evidence increasingly supports the idea that temporal disruptions in islet hormone release represent some of the earliest detectable abnormalities in the development of type 2 diabetes. However, despite decades of research, there remains no clear consensus on the mechanisms that govern hormone pulsatility within the portal circulation [4–6]. This gap highlights the urgent need for a more mechanistic and temporally precise understanding of how intra-islet communication governs the oscillatory nature of hormone output.

### Beyond the islet: A systemic view of pulsatility

Pulsatility is a core characteristic that runs through all levels of biology—from the tiniest atomic vibrations and molecular reactions to gene expression patterns, the rhythmic behaviors of tissues, and organ system coordination [7,8]. It’s likely that various systems-endocrine, neural, and vascular-work together to shape hormone signals by integrating inputs from both cellular and subcellular levels [9–12].

The liver, as the first stop for hormones coming from the islets, is in a prime position to interpret the timing and content of these pulses. When this delivery system-especially its rhythmic nature-is disrupted, it throws off the liver’s ability to regulate glucose output. However, the underlying mechanisms remain unclear [1,3]. What’s worth considering is that even though islets are highly specialized micro-organs capable of generating their own secretory rhythms, they may not be the ones entirely in control-the pace of this physiological symphony might be set elsewhere.

### Paracrine signaling: Central or peripheral in glucoregulation?

Islets should not merely be seen as hormone-producing clusters but as dynamic, self-organizing networks. They possess intrinsic oscillatory capabilities driven by intracellular mechanisms-such as calcium signaling, metabolic fluxes, and ion channel activity-that generate rhythmic hormone output. These processes are stabilized and coordinated by intercellular communication and feedback loops. [13–16]. Within this network, intra-islet paracrine interactions act as the fine-tuning machinery for hormonal precision. These local interactions integrate systemic inputs and control the timing, amplitude, and composition of hormonal pulses delivered to the portal vein [17–20].

Recent findings highlight that this paracrine crosstalk helps set glycemic thresholds in both mice and humans—a key step in maintaining glucose balance [21,22]. This underscores the importance of local signaling in regulating blood sugar levels and suggests a potential link to conditions like prediabetes. In fact, mounting evidence points to disrupted paracrine communication as a possible trigger for the transition from normal glucose levels to prediabetes [23–26]. Yet, despite this, we still lack detailed mechanistic studies explaining how paracrine feedback shapes hormone pulsatility-a critical and overlooked gap in diabetes research.

### Limitations of current approaches: Is the model still fit for purpose?

Much of what we currently understand about intra-islet communication comes from experimental strategies that, while methodologically accessible, fail to capture the dynamic complexity intrinsic to oscillatory systems. Prevailing models often lean heavily on data from static incubation assays or similar setups with limited temporal resolutiontools that may be convenient but are poorly suited for probing the inherently time-sensitive secretory behavior of islet cells [17–20]. These methods can imply potential signaling relationships, but they fall short in resolving the fast, rhythmic fluctuations that define physiological hormone release in real time.

A substantial body of work has investigated the paracrine effects of a wide array of ligands-Acetylcholine, Glutamate, Serotonin, GABA, Epinephrine, Urocortin3, Ghrelin, ATP, Zn^2+^, and others-each adding a piece to the puzzle of how islet cells modulate one another’s function [27–34]. Additionally, the paracrine interplay among the islet’s core hormones-insulin, glucagon, and somatostatin-has been examined in depth [35–38]. Yet despite the breadth of this work, a persistent limitation remains without dynamic, temporally resolved assays, these findings offer only a partial view of a system that is inherently rhythmic and coordinated in time [35–38].

### The danger of incomplete models

Despite their utility, many current models of intraislet signaling fail to incorporate key physiological features, particularly those tied to cellular dynamics. One notable oversight is the exclusion of post-inhibitory rebound excitation responses-commonly referred to as Post-Inhibitory Responses (PIR). This phenomenon, well-documented in neural, cardiac, and other oscillatory systems [39–42], represents a fundamental mechanism through which cells can regain activity following inhibitory input. Its absence from islet models may obscure critical feedback loops and dynamic behaviors that are central to the generation and regulation of pulsatile hormone release.

### Toward experimental precision: Tools and technologies

Two studies stand out in offering mechanistic depth: those involving Urocortin3 and Ghrelin [27,28]. These ligands selectively activate delta cells via distinct GPCRs, triggering somatostatin release. Transcriptomic data supports this specificity, yet both studies relied predominantly on static assays-underscoring the need for dynamic analysis using real-time perifusion or microfluidic tools.

Recent technological innovations offer promising avenues to overcome these barriers. Optogenetic and chemogenetic platforms now enable targeted stimulation or inhibition of individual cell types with temporal precision [43–46]. Coupled with high-resolution perifusion systems and hormone-sensing biosensors, these tools allow dynamic interrogation of islet function under near-physiological conditions.

### The case for perifusion and microfluidic systems

Notably, perifusion systems offer resolution between 0.5–5 minutes per fraction, which is sufficient to detect the oscillatory hormone release patterns characteristic of healthy islets [47–49]. High-resolution perifusion of whole pancreas tissue or pancreas slices offers clear advantages over isolated islet models. These approaches preserve islet architecture, maintain native vasculature, and enable hormone secretion profiling that reflects true in vivo dynamics [50–52]. Microfluidic “islet-on-a-chip” systems go further, integrating multiple sensor arrays for real-time monitoring of secretory and signaling events [53–59]. Despite their promise, these tools remain underexploited in the field’s standard research workflow.

### Reframing the research agenda

To advance our understanding of islet biology, we must embrace both the complexity and the dynamic nature of intra-islet communication. We must also acknowledge the inadequacies of current models and experimental designs. Critical steps forward include:

1. Temporal Fidelity: Adopt perifusion or microfluidic systems for high-resolution, real-time analysis [47–49].
2. Cell-Specific Resolution: Leverage cell-targeted optogenetics or chemogenetics to probe causal mechanisms with precise cellular specificity [43,45]
3. Physiological Relevance: Emphasize in situ or ex vivo preparations that preserve vascular and paracrine microenvironments [50,51].
4. Functional Readouts: Prioritize direct hormone measurements over indirect proxies like Ca^2+^ flux unless clearly linked to secretion [54,56].
5. Systems Integration: Incorporate vascular and neural inputs into islet models, shifting from reductionist to integrative physiology [11] (Table 1).

## Conclusion

### Are we asking the right questions?

The current conceptual model of intra-islet signaling, while instructive, falls short in accounting for the dynamic, oscillatory, and systemic integration of islet output. By embracing dynamic, high-resolution, and integrative approaches, we can move toward a truly mechanistic understanding of intraislet signaling. The tools are here, and the field is well-positioned to dissect these complexities. The question remains: are we designing studies that truly reflect the physiology we aim to understand?

Our ability to meaningfully intervene in diabetes depends on shifting from descriptive to mechanistic insight—particularly into how intra-islet communication shapes hormone pulsatility. Early disruptions in this finely tuned system may offer diagnostic value before overt dysfunction emerges. Precision therapies will rely on targeting specific paracrine pathways, such as somatostatin and glucagon signaling, in ways that respect the native dynamics of the islet. As experimental models grow more sophisticated, so must our questions. Only then can we align our interventions with the complexity of the system we seek to restore.

## Figures and Tables

**Table 1:** Comparative evaluation of islet secretion assessment methods.

Feature	Static Incubation	Perifusion	Natural Ligand Stimulus-Response Coupling	Optogenetic/Chemogenetic Cell-Specific Stimulation
**Description**	Islets are placed in a stationary medium for a set time to assess hormone secretion.	Islets are continuously perfused with medium to measure dynamic secretion over time.	Uses physiological ligands (e.g., glucose, amino acids) to trigger hormone release via endogenous pathways.	Activates specific cell types using light or designer drugs to dissect intra-islet circuit control.
**Pros**	- Simple setup - High-throughput - Cost-effective	- Time-resolved data - Better mimics in vivo - Control over stimulus	- Preserves native pathways - Physiologically relevant - Easy to apply	- Cell-type specificity - Temporal precision - Dissects causal relationships
**Cons**	- No time resolution - Feedback accumulation - Poor for dynamics	- Technical complexity - Expensive - Low throughput	- Stimulates all responsive cells - Indirect effects - Lacks specificity	- Requires genetic tools - Specialized equipment - Depends on targeting efficiency
**Temporal Resolution**	Poor	High	Moderate to high (depends on setup)	High (ms to min scale)
**Cell Specificity**	None	None	Low	High
**Mechanistic Insight**	Limited	Improved (temporal)	Moderate—pathway-level only	High—cell-type–resolved mechanisms
**Physiological Relevance**	Moderate	High	High	Variable (depends on targeting fidelity)
**Throughput**	High	Low to medium	Medium to high	Low to medium
**Equipment Needs**	Minimal	Specialized perifusion system	Standard lab setup	Optogenetics: light delivery Chemogenetics: viral tools, DREADDs
**Cost**	Low	High	Low to moderate	High
